# Predictive value of neutrophil-to-lymphocyte ratio on hospital outcomes of patients with community-acquired pneumonia

**DOI:** 10.1371/journal.pone.0348518

**Published:** 2026-05-29

**Authors:** Zakir Hossion, Zabir Hossain, Fatema Islam, Sharmin Yasmin

**Affiliations:** Dhaka Medical College Hospital, Dhaka, Bangladesh; Azienda Ospedaliero Universitaria Careggi, ITALY

## Abstract

**Background:**

Community-acquired pneumonia (CAP) remains a leading cause of hospitalization and mortality worldwide, particularly in low- and middle-income countries. Risk stratification is essential for guiding treatment decisions, yet commonly used biomarkers such as C-reactive protein and procalcitonin are costly and not always readily available. The neutrophil-to-lymphocyte ratio (NLR), a simple index derived from routine blood counts, has been proposed as a cost-effective prognostic marker. This study aimed to evaluate the predictive value of NLR on hospital outcomes in patients with CAP.

**Methods:**

A prospective observational study was conducted in the Department of Medicine at Dhaka Medical College Hospital, Bangladesh, between July 2023 and June 2024. One hundred adult patients with CAP were enrolled using purposive sampling. Demographic, clinical, and laboratory data were recorded at admission, including complete blood counts and CURB-65 scores. NLR was calculated, with ≥7.12 defined as elevated. Patients were followed for outcomes including mortality, oxygen requirement, ICU admission, and length of hospital stay. Statistical analyses included chi-square tests, logistic regression, and ROC curve analysis.

**Results:**

The mean age of participants was 53.7 ± 10.9 years, and 62% were male. An elevated NLR (≥7.12) was present in 67% of patients and was significantly associated with mortality (p = 0.027), oxygen requirement (p < 0.001), ICU admission (p < 0.005), and prolonged hospital stay (p < 0.001). Regression analysis confirmed NLR ≥ 7.12 is associated with prolonged stay (OR: 15.235; 95% CI: 4.613–50.316; *p* < 0.001), oxygen requirement (OR: 1.846; 95% CI: 1.415–2.408; *p* < 0.001), ICU transfer (OR: 5.172; 95% CI: 1.289–20.747; *p* < 0.001), and mortality (OR: 7.704; 95% CI: 1.962–61.690). ROC analysis showed moderate discriminatory ability for prolonged stay (AUC: 0.762) and modest predictive performance for oxygen need, ICU admission, and mortality.

**Conclusion:**

Elevated NLR is strongly associated with adverse outcomes in CAP, including mortality, ICU admission, oxygen requirement, and prolonged hospitalization. Given its simplicity and accessibility, NLR may serve as a valuable tool for early risk stratification in resource-limited settings.

## Introduction

Community-acquired pneumonia (CAP) is an acute respiratory infection involving the lung parenchyma that is acquired from a community setting. It remains one of the leading causes of hospitalization and mortality worldwide, particularly in low- and middle-income countries like Bangladesh [1]. Despite progress in diagnosis and management, including supportive care and antibiotic therapies, the mortality rate of CAP has not seen a significant decline in recent decades [2]. The burden of CAP is compounded by underlying comorbidities such as diabetes, tuberculosis, malignancy, and immunosuppression, which increase both incidence and severity. Studies have shown that up to 14% of hospitalized CAP patients succumb during their hospital stay, with factors such as drowsiness on admission and oxygen saturation below 90% associated with poor outcomes [1]. The etiological agents of CAP are well-documented and include both bacterial and viral pathogens, with *Streptococcus pneumoniae*, *Mycoplasma pneumoniae*, *Haemophilus influenzae*, and influenza viruses being among the most frequently identified [3]. However, bacterial cultures are not routinely performed in many resource-limited healthcare settings in developing countries, making it difficult to distinguish bacterial from viral causes. This dilemma often leads to the unnecessary use of antibiotics and contributes to the growing threat of antimicrobial resistance [4].

The clinical diagnosis of CAP is further complicated by variable microbial patterns based on age, comorbidities, and disease severity. For instance, *Streptococcus pneumoniae* is consistently identified across different patient groups, including outpatients, hospitalized patients, and those who died from the disease [5]. In countries like Bangladesh, comorbidities such as tuberculosis, diabetes mellitus, chronic steroid use, cancer, and HIV further amplify the disease burden, posing additional challenges for timely diagnosis and appropriate clinical management [1]. Despite advancements in imaging and microbiological testing, clinicians continue to rely on clinical judgment and laboratory markers to assess disease severity and prognosis [6]. Traditional biomarkers like C-reactive protein (CRP) and procalcitonin are commonly used in conjunction with clinical scores for risk stratification [7]. However, these markers are expensive, not readily available in all settings, and do not always yield satisfactory predictive value. This highlights the need for simpler, cost-effective, and reliable biomarkers to guide treatment decisions, particularly in resource-limited settings.

The neutrophil to lymphocyte ratio (NLR), derived from the routine complete blood count (CBC) parameters, has emerged as a promising candidate in this regard. The NLR reflects the balance between neutrophil elevation (a marker of inflammation) and lymphocyte suppression (a marker of physiological stress or immune suppression) under pathological stress [8]. Several studies have suggested that a high NLR is associated with worse outcomes in various conditions, including cancer, myocardial infarction, and critical illness [9]. In the context of CAP, one study found that the median NLR was significantly higher among patients who died during hospitalization (11.96) compared to survivors (4.19), and an NLR cut-off of 7.12 predicted in-hospital mortality with a sensitivity of 82.6% and specificity of 72.2% [9]. Other studies have similarly highlighted the potential of NLR to predict various clinical outcomes, such as the need for oxygen supplementation, ICU admission, and prolonged hospital stay [10,11]. The simplicity and accessibility of NLR make it especially useful in under-resourced settings like upazila-level hospitals in Bangladesh, where access to advanced diagnostic tools is limited. NLR could serve as a rapid screening tool to identify high-risk patients, inform early empirical therapy, facilitate timely decision-making, optimize resource utilization, reduce treatment delays, and potentially improve clinical outcomes.

Despite growing evidence on the prognostic role of NLR in CAP, data from resource-limited settings like Bangladesh remain limited. This study was therefore designed to evaluate the predictive value of NLR on hospital outcomes in patients with community-acquired pneumonia. The primary objective was to determine whether NLR can serve as a reliable predictor of adverse hospital outcomes, including mortality, length of stay, oxygen requirement, and need for intensive care. Secondary objectives included describing the socio-demographic characteristics of CAP patients, assessing their complete blood counts, calculating their NLR values, and evaluating the diagnostic accuracy of NLR for predicting hospital outcomes. By addressing these objectives, the study aims to contribute to the existing body of knowledge on CAP management. If validated, NLR could become an essential tool for risk stratification and clinical decision-making in CAP, particularly in resource-limited settings. This could ultimately lead to more effective, timely, and targeted interventions, reducing the morbidity and mortality associated with community-acquired pneumonia.

## Method

### Study design & setting

This prospective observational study was conducted in the Department of Medicine at Dhaka Medical College Hospital, Dhaka, Bangladesh, from July 2023 to June 2024. The study population comprised adult patients diagnosed with community-acquired pneumonia (CAP) admitted to the hospital. CAP was defined using Infectious Diseases Society of America criteria, supported by chest X-ray or thoracic computed tomography findings. A purposive non-randomized sampling technique was employed. Inclusion criteria included adult (aged > 18 years) diagnosed with severe CAP and willing to participate in the study. Patients with nosocomial pneumonia, ventilator-associated pneumonia, pulmonary tuberculosis, active malignancy, immunosuppression, HIV infection, pulmonary embolism, or those receiving palliative care were excluded.

The sample size was calculated using the standard formula:


$$\begin{array}{c} n=\frac{z^{\text{2}}pq}{d^{\text{2}}} \end{array}$$

Where:

- *Z* = 1.96 (standard normal deviate at 95% confidence)
- *p* = 0.49 (based on a previous study showing 49.2% pneumonia among hospitalized infection cases) [12]
- *q* = 1 – p = 0.51
- *d* = 0.10 (acceptable margin of error)

$$\begin{array}{c} \text{n}=\;\{{\left(\text{1}.\text{9}\text{6}\right)}^{\text{2}}\times \text{0}.\text{4}\text{9}\times \text{0}.\text{5}\text{1}\}/{\left(\text{0}.\text{1}\text{0}\right)}^{\text{2}}=\text{9}\text{5}.\text{9} \end{array}$$

To account for potential dropouts and to align with the study duration, a total of 100 patients were enrolled.

### Data collection procedure

After obtaining ethical approval, eligible patients with CAP were enrolled based on inclusion and exclusion criteria. Informed written consent was obtained prior to enrollment. CAP was diagnosed based on clinical features and radiological findings (chest X-ray or thoracic CT) according to the Infectious Diseases Society of America (IDSA) guidelines. A structured and pre-tested questionnaire was developed through a literature review. It included socio-demographic characteristics, clinical findings, laboratory values, and treatment outcomes. Each patient was assigned a unique ID. Baseline data, including age, sex, comorbidities, and clinical presentation, were collected using a case record form (CRF). The CURB-65 score was calculated at admission. Blood samples were collected on admission to measure complete blood count, including total leukocyte count, neutrophil count, and lymphocyte count using an automated hematology analyzer. NLR was calculated by dividing the absolute neutrophil count by the absolute lymphocyte count. A cut-off value of 7.12 was used based on previous study by Yang *et al.* [9]. Patients were monitored throughout their hospital stay. Clinical outcomes, including the need for oxygen support, ICU referral, length of hospital stay, and in-hospital mortality, were recorded. Each patient was followed up on the 3rd, 7th, and 10th day until discharge or death.

### Data processing and statistical analysis

Collected data were compiled, checked for consistency, and analyzed using IBM SPSS Statistics for Windows, Version 26.0. Continuous variables were expressed as mean ± standard deviation (SD) or median, while categorical variables were summarized as frequencies and percentages. Chi-square tests compared proportions, and univariate logistic regression calculated odds ratios. Receiver operating characteristic (ROC) curves evaluated NLR’s predictive accuracy, with AUC interpreted as follows: 0.50–0.59 (no value), 0.60–0.69 (poor), 0.70–0.79 (moderate), 0.80–0.89 (good), and 0.90–1.00 (excellent). A p-value <0.05 was considered statistically significant.

### Ethical considerations

Ethical clearance was obtained from the Research Review Committee and Ethical Review Committee of Dhaka Medical College. All participants provided informed consent, and data confidentiality was maintained throughout the study.

## Result

### Baseline characteristics

A total of 100 patients diagnosed with community-acquired pneumonia (CAP) were enrolled in the study. The mean age was 53.73 ± 10.86 years, with the majority (55%) falling between 45 and 64 years of age. Males comprised 62% of the study population, while females accounted for 38%. Smoking was prevalent, with 60% of patients having a history of smoking. A wide range of comorbidities was observed. Cardiovascular disease was the most common (30%), followed by chronic obstructive pulmonary disease (28.75%), diabetes mellitus (20%), chronic kidney disease (8.75%), and cardiac failure (7.5%). Other comorbid conditions were present in 5% of the patients. The mean systolic and diastolic blood pressures were 79.85 ± 12.84 mmHg and 57.75 ± 12.84 mmHg, respectively. The average CURB-65 score on admission was 2.38 ± 1.14, suggesting that most patients presented with moderate to severe pneumonia. Laboratory findings revealed a mean total white blood cell count of 14,542.97 ± 2,785.24 cells/cumm. The mean neutrophil percentage was 79.75 ± 5.49%, while the lymphocyte percentage averaged 10.73 ± 3.06%. The resulting mean neutrophil-to-lymphocyte ratio (NLR) was 7.97 ± 2.18. Based on a cut-off value of 7.12, 67% of patients had a high NLR (≥7.12), and 33% had a low NLR (<7.12) (Table 1).

**Table 1:** Baseline characteristics of the participants.

Variables	Frequency
**Age**	
Mean (SD)	53.73 (10.86)
**Age group**	
35-44 years	22 (22%)
45-54 years	27 (27%)
55-64 years	28 (28%)
>64 years	23 (23%)
**Sex**	
Female	38 (38%)
Male	62 (62%)
**Smoking habit**	
No	40 (40%)
Yes	60 (60%)
**Comorbidities**	
Cardiovascular disease	24 (30%)
COPD	23 (28.75%)
Diabetes	16 (20%)
Chronic kidney disease (CKD)	7 (8.75%)
Cardiac failure	6 (7.5%)
Others	4 (5%)
**Blood pressure (mmHg)**	
Systolic (Mean (SD))	79.85 (12.84)
Diastolic (Mean (SD))	57.75 (12.84)
**Laboratory parameters**	
WBC count (/cumm) (Mean (SD))	14542.97 (2785.24)
Neutrophil (%) (Mean (SD))	79.75 (5.49)
Lymphocyte (%) (Mean (SD))	10.73 (3.06)
NLR (Mean (SD))	7.97 (2.18)
**NLR category**	
≥ 7.12	67 (67%)
<7.12	33 (33%)
**CURB65 score**	
Mean (SD)	2.38 (1.14)

The study demonstrated a significant association between elevated neutrophil-to-lymphocyte ratio (NLR) and adverse hospital outcomes in patients with community-acquired pneumonia. Among those who died during hospitalization, 92.9% had an NLR ≥ 7.12, compared to only 7.1% with an NLR < 7.12 (*p* = 0.027), indicating a strong link between high NLR and mortality. Similarly, oxygen requirement was significantly higher among patients with elevated NLR, with 91.9% of oxygen-dependent patients having NLR ≥ 7.12 compared to 52.4% among those who did not require oxygen (*p* < 0.001). A comparable trend was observed for ICU admission, where 91.3% of ICU-admitted patients had a high NLR, as opposed to 59.7% among those not requiring intensive care (*p* < 0.005). Length of hospital stay was also significantly associated with NLR levels; 90.2% of patients with stays ≥7 days had NLR ≥ 7.12, whereas only 37.8% of patients with shorter stays (<7 days) had high NLR (*p* < 0.001) (Table 2).

**Table 2:** Association between NLR and hospital outcomes.

NLR	Hospital outcome		p value
**Death**			
	Yes (n = 14)	No (n = 86)	
<7.12	1 (7.1%)	32 (37.2%)	0.027
≥7.12	13 (92.9%)	54 (62.8%)	
**Oxygen requirement**			
	Yes (n = 37)	No (n = 63)	
<7.12	3 (8.1%)	30 (47.6%)	<0.001
≥7.12	34 (91.9%)	33 (52.4%)	
**ICU need**			
	Yes (n = 23)	No (n = 77)	
<7.12	2 (8.7%)	31 (40.3%)	<0.005
≥7.12	21 (91.3%)	46 (59.7%)	
**Length of stay**			
	<7 days (n = 45)	≥7 days (n = 41)	
<7.12	28 (62.2%)	4 (9.8%)	<0.001
≥7.12	17 (37.8%)	37 (90.2%)	

**Fig 1:**
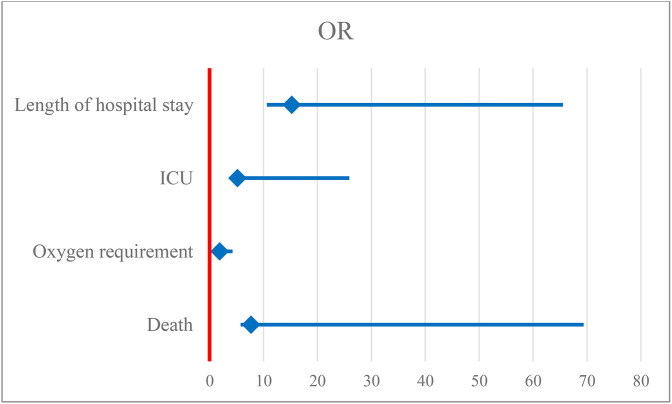
Forest plot showing the Odds ratio of NLR for predicting the hospital outcome of the study subjects.

Our study demonstrated that a neutrophil-to-lymphocyte ratio (NLR) of ≥7.12 was significantly associated with adverse clinical outcomes in patients with community-acquired pneumonia (CAP). Patients with elevated NLR had markedly higher odds of prolonged hospital stay (OR: 15.235; 95% CI: 4.613–50.316; *p* < 0.001), indicating a strong association with increased duration of illness and resource utilization. Similarly, the odds of requiring supplemental oxygen were significantly greater among those with high NLR (OR: 1.846; 95% CI: 1.415–2.408; *p* < 0.001), suggesting that systemic inflammation reflected by NLR is associated with respiratory compromise. Furthermore, elevated NLR was strongly associated with increased likelihood of ICU admission (OR: 5.172; 95% CI: 1.289–20.747; *p* < 0.001), underscoring its potential as a marker of disease severity. Notably, high NLR also predicted in-hospital mortality (OR: 7.704; 95% CI: 1.962–61.690), confirming its prognostic value in identifying patients at increased risk of death. (Fig 1)

The Table 3 summarizes the diagnostic performance of an elevated neutrophil-to-lymphocyte ratio (NLR ≥ 7.12) in predicting adverse hospital outcomes in patients with community-acquired pneumonia. NLR showed high sensitivity across all outcomes—91.9% for oxygen requirement, 91.3% for ICU admission, 92.9% for mortality, and 90.2% for prolonged hospital stay—indicating its strong ability to identify at-risk patients. The negative predictive values (NPVs) were also notably high (87.5%–97.0%), suggesting that a low NLR reliably excluded poor outcomes. However, specificity and positive predictive value (PPV) were lower across outcomes, particularly for mortality (specificity 37.2%, PPV 19.4%), limiting NLR’s ability to confirm adverse outcomes when elevated. Statistically significant associations were observed for oxygen need, ICU admission, and prolonged stay (all *p* < 0.05), while the association with mortality did not reach significance (*p* = 0.072). Overall, elevated NLR is a useful tool for early risk stratification, particularly for ruling out severe outcomes in CAP patients.

**Table 3:** Diagnostic accuracy of NLR for predicting hospital outcomes in patients with community-acquired pneumonia.

Hospital outcome	Sensitivity	Specificity	PPV	NPV	p value
Oxygen requirement	91.9%	47.6%	50.7%	90.9%	<0.001
ICU need	91.30%	40.30%	31.30%	93.90%	0.022
Mortality	92.90%	37.20%	19.40%	97.00%	0.072
Increased length of hospital stays	90.20%	62.20%	68.50%	87.50%	<0.001

**Fig 2:**
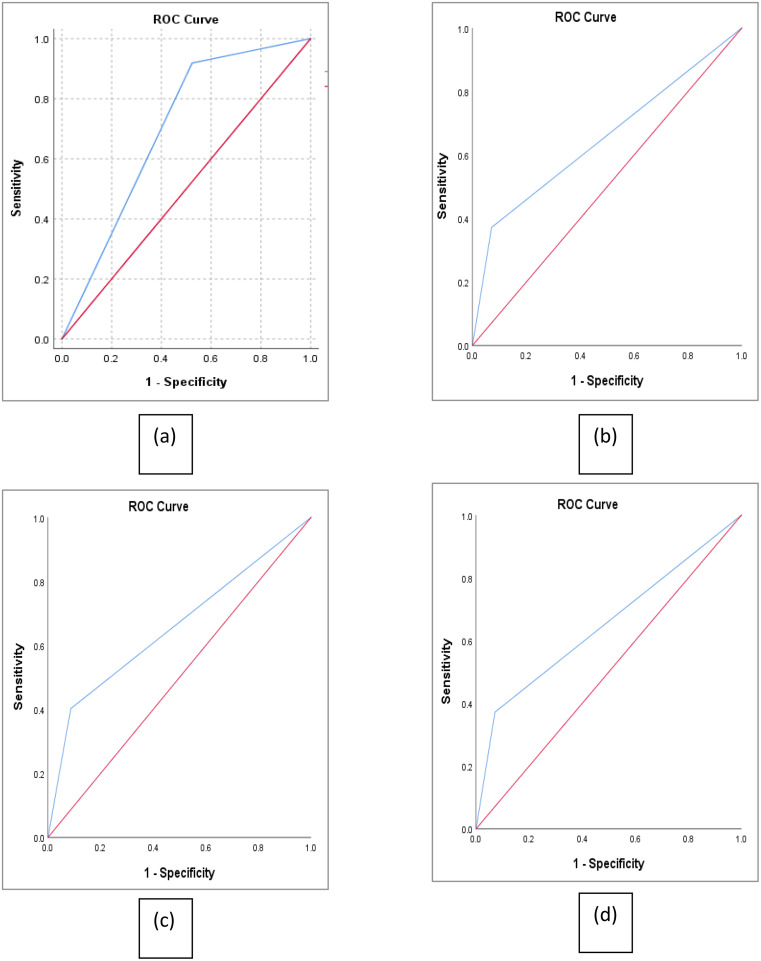
Receiver operating characteristic curve (ROC) analysis of NLR in predicting (a) oxygen requirement, (b) hospital mortality, (c) ICU need, (d) increased length of hospital stay in CAP.

Receiver operating characteristic (ROC) curve analysis demonstrated that the neutrophil-to-lymphocyte ratio (NLR) had varying degrees of discriminatory power for predicting adverse hospital outcomes in community-acquired pneumonia. The area under the curve (AUC) for NLR was 0.698 for predicting oxygen requirement (a), 0.650 for mortality (b), 0.658 for ICU transfer (c), and 0.762 for prolonged hospital stay (d). These findings suggest that NLR ≥ 7.12 had some discriminatory ability for predicting oxygen need, ICU transfer, and mortality, and a moderate discriminatory value for identifying patients at risk of extended hospitalization (Fig 2).

## Discussion

This study identified that an elevated neutrophil-to-lymphocyte ratio (NLR ≥ 7.12) is strongly associated with adverse outcomes in patients with community-acquired pneumonia (CAP), including prolonged hospital stay, increased oxygen requirement, ICU admission, and mortality. These findings suggest that patients with elevated NLR are significantly more likely to experience severe disease and poor clinical outcomes. Ju et al. (2019) similarly reported that elevated NLR (≥6.161) was associated with early clinical instability (OR 3.44; 95% CI 1.74–6.80), reinforcing the role of NLR as a prognostic marker in CAP [13].

Comparing these results with larger and more recent studies reveals some differences in effect size but consistent trends. For example, a large multicenter Australian study analyzing 7,862 CAP patients found that NLR > 12 was associated with prolonged length of stay (incidence rate ratio 1.11), increased ICU admission risk (adjusted OR 1.41), and higher in-hospital mortality (adjusted OR 1.27), all statistically significant but with more modest effect sizes than those observed in the current study [14]. The discrepancy in odds ratios may be due to differences in NLR cutoff values (7.12 vs. 12), patient populations, sample sizes, and adjustment for confounders such as age, comorbidities, and severity scores. Systematic reviews and meta-analyses further support the prognostic value of NLR in CAP. A comprehensive review of 3,340 patients reported that NLR cutoff values above 10 predicted mortality more accurately than traditional markers like C-reactive protein and CURB-65 in most included studies [8]. However, some studies, such as a single-center retrospective cohort, found that after adjusting for Pneumonia Severity Index (PSI), NLR was not associated with mortality, suggesting that NLR alone may not fully capture risk without considering established clinical scores [8]. Additionally, studies indicate that NLR correlates positively with clinical severity indices such as CURB-65 and Pneumonia Severity Index (PSI), and combining NLR with these scores modestly improves predictive power. For example, a retrospective study found that combining NLR with CURB-65 increased the AUC from 0.67 to 0.70 for predicting early clinical instability, suggesting a complementary role rather than a replacement [13].

The elevated NLR reflects neutrophil-driven tissue damage and systemic inflammation, which can exacerbate lung injury and multi-organ dysfunction. Simultaneously, lymphopenia may impair pathogen clearance and immune homeostasis, increasing susceptibility to complications and death. This dual mechanism explains why patients with high NLR are more likely to require oxygen therapy, ICU care, prolonged hospitalization, and have higher mortality. Furthermore, the predictive value of NLR has been demonstrated across diverse populations, including pediatric CAP patients, where elevated NLR was associated with markedly increased mortality risk (OR up to 67.98) after adjusting for confounders. This underscores the utility of NLR as a universally applicable, inexpensive, and readily available biomarker for early risk stratification.

The diagnostic performance of the neutrophil-to-lymphocyte ratio (NLR) reported in this study indicates that NLR is a highly sensitive but moderately specific marker. The high sensitivity and negative predictive values suggest that NLR is especially useful as a rule-out test, effectively identifying patients unlikely to experience these adverse outcomes. However, its modest specificity and positive predictive values imply a considerable rate of false positives, limiting its utility as a standalone diagnostic tool.

These findings are broadly consistent with the literature. For instance, Zhong et al. (2021) reported a sensitivity of 84.8% and specificity of 74.8% for NLR in predicting mortality among elderly CAP patients, indicating somewhat better specificity than in the current study but still reflecting the trade-off between sensitivity and specificity common to inflammatory biomarkers [15]. Similarly, a large Australian cohort study analyzing 7,862 CAP patients found that while elevated NLR (> [12]) was associated with increased ICU admission and mortality, its predictive accuracy (AUC ~ 0.58) was modest and inferior to established clinical scores like CURB-65 (AUC ~ 0.68), and adding NLR to CURB-65 did not significantly improve prognostication [14]. Systematic reviews corroborate that NLR is a simple, inexpensive, and easily measurable biomarker with promising prognostic value in CAP, often outperforming traditional inflammatory markers such as C-reactive protein or white blood cell count. However, its moderate specificity limits its role as a definitive diagnostic tool. Instead, it is best applied as part of a multimodal assessment, complementing clinical severity scores and other laboratory parameters to enhance risk stratification. The relatively lower specificity observed in this study compared to some others may be influenced by differences in patient populations, NLR cutoff values (7.12 here versus higher thresholds like 10 or 12 in other studies), timing of measurement, and comorbidities affecting baseline inflammatory status. Moreover, factors such as steroid use or concomitant infections can alter neutrophil and lymphocyte counts, potentially confounding NLR’s accuracy.

However, specificity is lower because elevated NLR is not exclusive to severe CAP; it can also occur in various other inflammatory, infectious, or stress-related conditions. For example, smokers, patients with chronic diseases, or those with mild infections may have elevated neutrophils or suppressed lymphocytes for reasons unrelated to CAP severity, leading to false positives. This reduces NLR’s ability to “rule in” severe outcomes with high precision. Moreover, factors like corticosteroid use, viral infections, or other comorbidities can alter neutrophil and lymphocyte counts independently of pneumonia severity, further lowering specificity. This pattern is supported by a systematic review of 3,340 CAP patients, which reported NLR sensitivities ranging from 56.4% to 78.3% and specificities between 51.6% and 66.8% at a cutoff of 10, with higher cutoffs (e.g., 11.2) improving both sensitivity and specificity but often at the cost of missing some cases [8]. Another study found NLR sensitivity of 80% and specificity of 70% for predicting 30-day mortality, with performance inferior to but complementary to the Pneumonia Severity Index (PSI) [10]. These findings illustrate that while NLR is a sensitive marker reflecting systemic inflammation, its moderate specificity limits its use as a standalone diagnostic tool.

The reported ROC curve analysis showing moderate discriminatory power of NLR for prolonged hospital stay and some predictive value for oxygen requirement, mortality, and ICU transfer aligns with findings from prior studies, supporting NLR as a useful, accessible prognostic biomarker in community-acquired pneumonia (CAP). These AUC values indicate that NLR performs reasonably well in distinguishing patients at risk of adverse outcomes, particularly for length of stay, though its accuracy is less robust for mortality and ICU admission. Comparatively, de Jager et al. (2012) reported an AUC of 0.701 for mortality prediction, while Ju et al. (2019) found an AUC of 0.662 for early clinical instability, both consistent with the current study’s moderate predictive ability [13,16]. However, larger cohort studies, such as a recent Australian multicenter analysis of 7,862 CAP patients, found that although elevated NLR (> [12]) was associated with prolonged length of stay, ICU admission, and mortality, its predictive accuracy was modest (AUC ~ 0.58 for mortality), and it did not improve the CURB-65 score’s performance (AUC ~ 0.68) when combined [14]. This suggests that while NLR is a valuable marker of systemic inflammation and disease severity, it may not surpass or significantly enhance established clinical risk scores. Meta-analyses further reinforce these conclusions. A pooled analysis of over 5,000 CAP patients showed admission NLR had a moderate AUC of approximately 0.71 for 30-day mortality prediction, improving substantially when measured 3–5 days after admission (AUC ~ 0.88), highlighting the potential benefit of serial NLR measurements over a single time point. This dynamic assessment may better capture evolving inflammatory responses and clinical trajectory [17].

In this study, the mean age of patients with community-acquired pneumonia (CAP) was 53.73 ± 10.86 years, with the largest proportion (28%) falling within the 55–64 years age group. These findings align with previous research by Simonetti et al. (2014) and Cillóniz et al. (2018), who reported that while CAP can occur at any age, both incidence and mortality risk increase with advancing age [6,18]. Simonetti et al. further explained that the decline in pulmonary host defenses in the elderly, including diminished function of antigen-presenting cells such as natural killer cells, macrophages, and neutrophils, contributes to this increased vulnerability [18]. Regarding sex distribution, 62% of the study participants were male and 38% female, resulting in a male-to-female ratio of approximately 1.6:1. This male predominance is consistent with findings from Barbagelata et al. (2020), who concluded that CAP tends to be more severe in males, leading to higher mortality, especially among older patients [19]. Similarly, Quero et al. (2017) observed that 64% of hospitalized CAP patients were men and noted that males often have more toxic habits and comorbidities, which may contribute to worse outcomes [20]. In the present study, 60% of patients were smokers, with 58.8% of male patients reporting tobacco use. This is supported by Baskaran et al. (2019), who described how smoking impairs mucociliary clearance by increasing mucus production and damaging cilia, thereby facilitating bacterial adherence and colonization [21]. Almirall et al. (2014) emphasized that tobacco exposure, including passive smoking, significantly increases susceptibility to bacterial lung infections, particularly those caused by Streptococcus pneumoniae, the most common pathogen associated with CAP in smokers [22]. Comorbidities were prevalent among the study population, with 24% having cardiovascular disease, 23% chronic obstructive pulmonary disease (COPD), 16% diabetes mellitus, 7% chronic kidney disease, 6% chronic cardiac failure, and 4% other chronic pulmonary diseases. These findings are consistent with Eldaboosy et al. (2015) and Chalmers et al. (2008), who reported that diabetes, renal impairment, heart failure, and prior cerebral insults are important risk factors for CAP [23,24]. The mean systolic and diastolic blood pressures recorded were 79.85 ± 12.84 mmHg and 57.75 ± 12.84 mmHg, respectively. These values are somewhat lower than those reported by Curtain et al. (2013) and Aziz et al. (2016), who found mean systolic pressures of 101.86 ± 19.38 mmHg and 93.1 ± 20.7 mmHg, and diastolic pressures of 72.9 ± 14.41 mmHg and 49.4 ± 10.9 mmHg, respectively, possibly reflecting differences in patient severity or measurement timing. The mean CURB-65 score in this study was 2.38 ± 1.14, indicating moderate pneumonia severity. Carlos et al. (2023) reported a similar distribution, with over half of their patients scoring 3, and Aziz et al. (2016) observed a mean score of 3.1 ± 1.3, supporting the relevance of CURB-65 as a severity assessment tool in CAP [25,26].

However, this study has several limitations. First, the relatively small sample size and limited number of outcome events may reduce statistical power and increase the risk of imprecise estimates. Second, the single-center design may limit the generalizability of the findings to other settings and populations. Third, the use of purposive sampling may introduce selection bias. Finally, the NLR cut-off value was adopted from prior studies and may not represent the optimal threshold for this population.

## Conclusion

This study demonstrates that an elevated neutrophil-to-lymphocyte ratio (NLR ≥ 7.12) is strongly associated with adverse outcomes in patients with community-acquired pneumonia, including prolonged hospital stay, increased oxygen requirement, ICU admission, and mortality. NLR showed high sensitivity and negative predictive value, making it a useful tool for early risk stratification, particularly for ruling out severe outcomes. Although its specificity was modest, the simplicity, low cost, and wide availability of NLR make it a valuable prognostic biomarker in resource-limited settings where advanced diagnostic tests are not feasible. Incorporating NLR into clinical assessment alongside established severity scores may enhance decision-making, optimize resource utilization, and improve patient outcomes.
